# Sub-minimum inhibitory concentrations of colistin and polymyxin B promote *Acinetobacter baumannii* biofilm formation

**DOI:** 10.1371/journal.pone.0194556

**Published:** 2018-03-19

**Authors:** Yoshinori Sato, Yuka Unno, Tsuneyuki Ubagai, Yasuo Ono

**Affiliations:** Department of Microbiology and Immunology, Teikyo University School of Medicine, 2-11-1 Kaga, Itabashi-ku, Tokyo, Japan; University of Cambridge, UNITED KINGDOM

## Abstract

We investigated the numbers of planktonic and biofilm cells and the expression levels of genes encoding efflux pumps and biofilm-related proteins in 10 clinical isolates of multi-drug resistant *Acinetobacter baumannii* (MDRA) as well as in its standard strain ATCC 19606 in the presence of colistin (CST), polymyxin B (PMB), minomycin (MIN), and tigecycline (TGC) at their respective sub-MICs. The number of planktonic and biofilm cells of ATCC 19606 decreased in the presence of all aforementioned antibiotics in a dose-dependent manner. Cell number also decreased in two representative MDRA strains, R2 and R3, in the presence of MIN and TGC in a dose-dependent manner. In contrast, the number of biofilm cells in these two strains increased in the presence of CST, while they increased significantly in the presence of PMB in R2 only. Pearson correlation analysis revealed that the number of biofilm cells was positively and significantly correlated with the mRNA levels of genes encoding efflux pumps (*adeB* and *adeG*) and autoinducer synthase (*abaI*) in strain R2 and *adeB*, *adeG*, *adeJ*, poly-acetyl-glucosamine-porin (*pgaA*), and *abaI* in strain R3 in the presence of CST. It was positively and significantly correlated with the mRNA levels of genes encoding *adeB* in strain R2 and an outer membrane protein A (*ompA*) and biofilm-associated protein (*bap)* in strain R3 in the presence of PMB. These results provide valuable insights into the biofilm formation potency of clinical isolates of MDRA that depends on efflux pumps and biofilm-related genes and its regulation by antibiotics.

## Introduction

*Acinetobacter baumannii* is an important opportunistic pathogen associated with nosocomial infections, such as bacteremia, pneumonia, meningitis, urinary tract infections, and wound infections [1–3]. One of the main reasons for the recent increase in scientific interest in *A*. *baumannii* is the appearance of multi-drug resistant *A*. *baumannii* (MDRA) strains, which show resistance to almost all available antibiotics, including β-lactams, fluoroquinolones, tetracyclines, and aminoglycosides [3, 4]. Polymyxins and tigecycline (TGC) now remain the only effective antibiotics for the treatment of MDRA [5], although there have been reports of antibiotic resistance against these worldwide as well [6, 7]. Therefore, the recent increase in outbreaks of MDRA all over the world is a cause for concern [8, 9]. In addition, although *A*. *baumannii* is regarded as a low-virulence pathogen [4], recent studies have reported that *A*. *baumannii* shows several forms of pathogenicity, such as biofilm formation as well as adherence on and invasion of host cells [10–13], host cell death [14], and iron acquisition [15]. Therefore, *A*. *baumannii*, especially MDRA, has gradually gained importance as a human pathogen, particularly in hospital or clinical environments [8]. For the treatment of MDRA infection, polymyxins and minomycin (MIN) with its derivative, TGC, are used as active antibiotics [5].

Antibiotics are effective therapeutic agents against bacterial pathogens, when their concentrations become higher than the minimum inhibitory concentration (MIC) between consecutive doses. However, the administered concentration in various tissues usually becomes lower than the respective MIC for an antibiotic after a certain period of time following a dose. These concentrations of antibiotics below the MIC are defined as sub-minimum inhibitory concentrations (sub-MICs) [16]. Although the growth of an antibiotic-susceptible bacteria in the presence of antibiotics at sub-MICs is compromised as compared to its possible growth in the absence of antibiotics, the bacteria nonetheless continue to grow at sub-MICs [16, 17]. However, antibiotics at their sub-MICs can affect the morphology, surface properties, pathogenicity, and biofilm formation of bacteria [16, 17], while regulating gene transcription for normal bacterial homeostasis and virulence factors [16–19]. Notably, there have been numerous reports of antibiotic-induced biofilm formation [20].

Polymyxins (including polymyxin B (PMB) and colistin (CST)) are polycationic antimicrobial peptides and interact with the lipid-A moiety of anionic lipopolysaccharide (LPS) in gram-negative bacteria to cause disorganization of the outer membrane in bacteria, resulting in a rapid bactericidal effect [21]. Previous studies reported that polymyxins inhibited biofilm formation of *A*. *baumannii* and various other bacteria [22, 23], indicating suitable antibiotics for the treatment of bacterial infections. On the other hand, previous studies reported that biofilm formation in clinical isolates of *A*. *baumannii* was induced by imipenem (IPM), levofloxacin (LVX) and meropenem (MEM) at their respective sub-MICs [24, 25]. Additionally, He et al. demonstrated the induction of AdeFGH efflux pump in the presence of LVX and MEM at their sub-MICs and further highlighted the potential role of the AdeFGH efflux pump in the synthesis and transportation of quorum sensing (QS) molecules during biofilm formation [25]. Although biofilm formation in *A*. *baumannii* is associated with QS of its cells [26], these results suggest that antibiotics at their sub-MICs induce biofilm formation of *A*. *baumannii* associated with the mechanisms of QS. Moreover, biofilm formation in bacteria is positively correlated with the expression of several virulence factors, including the outer membrane protein, OmpA; the extracellular polysaccharide, poly-β-(1,6)-N-acetyl glucosamine (PNAG); type I pili; a homolog of the staphylococcal biofilm-associated protein (Bap); and the outer membrane protein, CarO [26]. However, there is relatively little literature available on the effects of antibiotics at their sub-MICs on biofilm formation and the expression of its related-genes of *A*. *baumannii*.

In this study, we have focused on the effects of polymyxins (CST and PMB), MIN, and TGC at their sub-MICs on the growth of planktonic clinical isolates of MDRA and their biofilm formation. We have further investigated the expression levels of genes encoding efflux pumps and biofilm-related proteins in these isolates in the presence of the same antibiotics at their respective sub-MICs with the aim of exploring the associations between the expression of relevant genes and biofilm formation in clinical isolates of *A*. *baumannii*.

## Materials and methods

### Bacterial strains and growth condition

Ten strains (R1-R10) of *A*. *baumannii* were isolated from the Teikyo University hospital during an infection outbreak that occurred around 2010. Isolations were carried out on CHROMagar™ *Acinetobacter* for 24 hours at 37°C. The isolates were streaked onto blood agar plates and cultivated for 24 hours to obtain monoclonal colonies. They were then identified as *A*. *baumannii* by DNA sequencing of a partial RNA polymerase β-subunit (*rpoB*) gene [27]. Additionally, the isolates were confirmed to be non-clonal isolates using pulsed-field gel electrophoresis (data not shown). After identification, these isolates were stored in glycerol stock at -80°C at the Department of Microbiology & Immunology, Teikyo University School of Medicine. Antimicrobial susceptibility testing was performed using all 10 strains of *A*. *baumannii* based on the MICs for IPM, AMK, and ciprofloxacin (CIP). The MICs of IPM, AMK, and CIP were >8, 16, and 2 mg/l, respectively, for these strains due to which they were identified as MDRA strains. The *A*. *baumannii* ATCC 19606 strain was used as a standard strain. These bacteria were cultured in Luria-Bertani (LB) broth (Becton, Dickinson and Company, MD, USA) for 16 hours at 37°C. The bacteria were then resuspended in LB broth at a concentration of 5×10^5^ cfu/ml, with the concentration being adjusted via optical density (OD) measurements at 595 nm. The cell suspensions thus obtained were used for various assays in the investigation.

### Determination of MICs

The MICs of CST, PMB, MIN, and TGC for *A*. *baumannii* were determined using a broth microdilution method with a starting inoculum of approximately 5×10^5^ cfu/ml of bacteria. The MIC was defined as the lowest concentration of antibiotic at which no bacterial growth was observed.

### Quantification of planktonic and biofilm cells

The quantification of planktonic and biofilm cells was performed in 24-well-plates. The bacteria were inoculated at a density of approximately 5×10^5^ cfu/ml in LB broth with antibiotics present at their sub-MICs (0, 1/4, and 1/2 MIC), where 0 MIC was considered a control. They were incubated for 24 hours at 37°C. After incubation, the supernatant from each well was transferred to a corresponding well in a new plate and OD was measured at 595 nm. This was used to define the number of planktonic cells. Any biofilm cells remaining in each well were washed three times with sterile distilled water, followed by staining with 1% crystal violet (Merck, Darmstadt, Germany) solution for 15 minutes. The cells were then washed three times with sterile distilled water and air-dried for an hour. Stained biofilm cells were de-stained using 95% ethanol; the OD was measured again at 595 nm. This was used to define the number of biofilm cells. The results were expressed as percentage of control determined by the equation: [(A/A_0_) ×100], where A_0_ and A are absorbance i.e., OD, at 0 MIC and 1/4 or 1/2 MIC of antibiotics, respectively.

### RNA extraction and quantitative real-time polymerase chain reaction (qPCR)

Total RNA of *A*. *baumannii* was extracted using an RNeasy Protect Bacteria Mini kit (Qiagen, Tokyo, Japan). To analyze the efflux pump and biofilm-related gene expression levels in *A*. *baumannii*, total RNA was extracted from the bacteria after 16 hours of incubation. Harvested RNA samples were quantified using the NanoDrop spectrophotometer (Thermo Fisher Scientific, MA, USA). Total RNA was reverse-transcribed to cDNA using PrimeScript™ 1^st^ strand cDNA Synthesis Kit (Qiagen). To analyze the mRNA levels of all genes, cDNA was amplified using the SYBR Green PCR Master Mix (Thermo Fisher Scientific, MA, USA) with consensus primers for detecting *adeB*, *adeG*, *adeJ*, *ompA*, *bap*, *pgaA*, *abaI* and *rpoB*. The primer sequences are listed in Table 1. The gene, *rpoB*, was used as an internal control for the quantification of *adeB*, *adeG*, *adeJ*, *ompA*, *bap*, *pgaAI*, and *abaI*. Real-time PCR was performed as follows: 40 cycles of denaturation at 95°C for 15 seconds, annealing at 62°C for 30 seconds, and extension at 72°C for 1 minute in each cycle. The amplified PCR products were quantitatively monitored using a StepOne Real-Time PCR System (Applied Biosystems, CA, USA). Fold changes in the mRNA expression were calculated by the 2^−ΔΔCt^ method using *rpoB* gene as an internal control [28]. The relative expression of each sample mRNA was evaluated relative to the control sample (0 MIC), which was assigned a value of 1 arbitrary unit.

**Table 1:** Primers used for real-time PCR.

Gene	Molecular function	Sequence	Reference
*adeB*	Inner membrane protein of AdeABC; efflux pump	F: GCTTTTCTACTGCACCCAAA R: CTTGCATTTACGTGTGGTGT	[25]
*adeG*	Inner membrane protein AdeFGH; efflux pump	F: TTCATCTAGCCAAGCAGAAG R: AGTAACCAGTGGCACTACAC	[25]
*adeJ*	Inner membrane protein AdeIJK; efflux pump	F: GGTCATTAATATCTTTGGC R: GGTACGAATACCGCTGTCA	[25]
*ompA*	Porin; biofilm formation, multidrug resistance	F: AACAAATCAAACATCAAAGACCAA R: GGTATTCAGATAATTTTTCAGCAACTT	[39]
*bap*	Biofilm-associated protein; biofilm maturation	F: CCTTGGTAACCACAGAGGGA R: TGACTGCATTGGTACCCTCC	[40]
*pgaA*	Protein that synthesize cell-associated poly-beta-(1–6)-N-acetylglucosamine (PNAG)	F: GCTGAAGCTCAAGATGTGGC R: ATGCAACCCGTACCAACTGA	[40]
*abaI*	Autoinducer synthase	F: AATGCCTATTCCCTGCTCAC R: ATTGCTTCTTGCAGAATTGC	[25]
*rpoB*	Reference gene	F: ATGCCGCCTGAAAAAGTAAC R: CGAGCGCCTACTGGAATTA	[25]

F, forward primer; R, reverse primer.

### Correlation analyses and statistics

The data obtained from mRNA expression analysis on efflux pumps and biofilm-related genes were expressed as means ± standard error of the mean (SEM) for three independent experiments performed in duplicates. Comparisons of numerical data were performed using Student’s *t* test and one-way analysis of variance (ANOVA), followed by the Dunnett’s multiple comparison test. Pearson correlation analysis was used to compare the mRNA levels of genes encoding efflux pump and biofilm-related proteins as well as the number of biofilm cells. In all analyses, a 2-tailed probability of < 5% (i.e. *p < 0.05) was considered statistically significant.

## Results

### Antibiotic susceptibilities of planktonic *A*. *baumannii* clinical isolates

The antibiotic susceptibilities of clinical isolates of MDRA and a standard strain, ATCC 19606, were determined in terms of MICs of antibiotics against the isolates’ planktonic cells. These MICs for representative (ATCC 19606, R2, and R3) and other strains investigated here are shown in Table 2 and S1 Table, respectively. All strains were sensitive to CST (≤4 μg/ml), PMB (≤2 μg/ml), and TGC (≤1 μg/ml). All strains, excluding R9, were sensitive to MIN (≤4 μg/ml) as well. The strain R9 exhibited intermediate resistance to MIN (≤8 μg/ml).

**Table 2:** MICs (μg/mL) of antibiotics against *A*. *baumannii* ATCC 19606 and the clinical isolates of MDRA.

		Antibiotics			
*A*. *baumannii*		Colistin (CST)	Polymyxin B (PMB)	Minocycline (MIN)	Tigecycline (TGC)
ATCC 19606		2	2	0.25	0.5
MDRA	R2	2	2	4	0.5
	R3	2	2	4	1

### Effect of antibiotics on the growth of planktonic *A*. *baumannii* clinical isolates

We analyzed the number of planktonic cells of *A*. *baumannii* after 24 hours of incubation *in vitro*. The number of planktonic cells for 6 strains (R1, R4, R5, R6, R7, and R8) were significantly higher than those for ATCC 19606, whereas the number of planktonic cells in the cultures of strains, R9 and R10, were significantly lower than those observed in the ATCC 19606 culture (S1A Fig). Planktonic cells of the strains, R2 and R3, showed almost the same number of cells as those of the strain, ATCC 19606. These results indicate that most of the clinical isolates of MDRA have high levels of growth.

**Fig 1:**
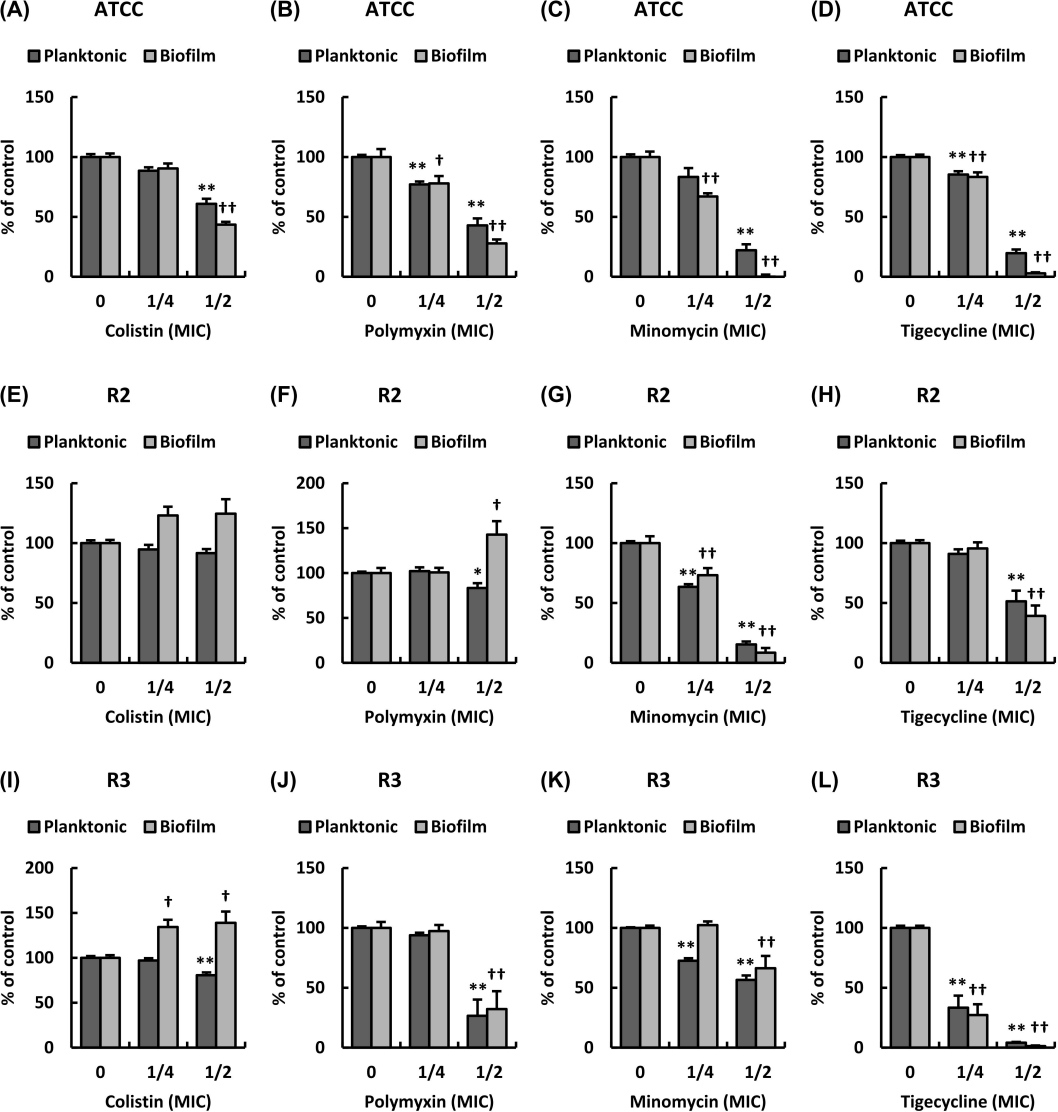
Effect of four antibiotics at sub-MICs on bacterial growth and biofilm formation of *A*. *baumannii*. Summarized results showing the ratio of planktonic and biofilm cells in strain ATCC 19606 cultured in LB broth with (A) CST, (B) PMB, (C) MIN and (D) TGC at sub-MICs. Summarized results showing the ratio of planktonic and biofilm cells in strain R2 cultured in LB broth with (E) CST, (F) PMB, (G) MIN and (H) TGC at sub-MICs. Summarized results showing the ratio of planktonic and biofilm cells in strain R3 cultured in LB broth with (I) CST, (J) PMB, (K) MIN and (L) TGC at sub-MICs. Dark gray and gray bars indicate the ratio of planktonic and biofilm cells in *A*. *baumannii*, respectively. Bar graph data are shown as the mean ± SEM (n = 6) of 3 independent experiments. Asterisks indicate statistically significant differences in the number of planktonic cells (**P< 0.01; *P< 0.05, non-treated bacteria *vs*. antibiotics-treated bacteria; One-way ANOVA). Crosses indicate statistically significant differences in the number of biofilm cells (^††^*P*<0.01; ^†^*P*<0.05, non-treated bacteria *vs*. antibiotics-treated bacteria; One-way ANOVA).

Furthermore, to investigate the effects of CST, PMB, MIN, and TGC at their sub-MICs on the growth of clinical isolates of MDRA, we analyzed the number of planktonic cells obtained for the isolates and ATCC 19606 after 24 hours of incubation in the presence of the aforementioned antibiotics at their sub-MICs. Representative data on the number of planktonic cells for strains, ATCC 19606, R2, and R3 are shown in Fig 1 and that for others are shown in S2 and S3 Figs. The number of planktonic cells for strains, ATCC 19606 and R10, significantly decreased in the presence of CST in a dose-dependent manner (Fig 1A and S3M Fig), whereas the number of cells for strains, R2 and R3, decreased slightly in the presence of CST in a dose-dependent manner (Fig 1E and 1I). The number of planktonic cells for strains, R7 and R9, decreased in the presence of CST at 1/2 MIC (S3A and S3I Fig), whereas the number of cells for strains, R1, R4, R5, R6, and R8, showed a 10% decrease in the presence of CST as compared to that in the absence of CST (S2A, S2E, S2I, S2M and S3E Figs). The number of planktonic cells for strains, ATCC 19606 and R10, significantly decreased in the presence of PMB in a dose-dependent manner (Fig 1B and S3N Fig). The number of cells for strains, R2, R3, R1, R4, R5, R7, R8, and R9, decreased in the presence of PMB at 1/2 MIC (Fig 1F and 1J) (S2B, S2F, S2J, S3B, S3F and S3J Figs). The number of planktonic cells for strain R6 in the presence of PMB was not altered compared to that in the absence of PMB (S2N Fig). The number of planktonic cells for strains ATCC 19606 and the 10 clinical isolates significantly decreased in the presence of MIN in a dose-dependent manner (Fig 1C, 1G and 1K) (S2C, S2G, S2K, S2O, S3C, S3G, S3K and S3O Figs). The number of planktonic cells for strains, ATCC 19606, R3, R1, R4, R5, R6, R8, and R9, significantly decreased in the presence of TGC in a dose-dependent manner (Fig 1D and 1L) (S2D, S2H, S2L, S2P, S3H and S3L Figs). The number of planktonic cells for strains, R2, R7, and R10, significantly decreased in the presence of TGC at 1/2 MIC (Fig 1H) (S3D and S3P Fig). These results indicate that five out of every ten MDRA isolates and one out of every ten isolates exhibit weak susceptibility to CST and PMB, respectively, at their sub-MICs; also, the clinical isolates of MDRA are susceptible to MIN and TGC at their sub-MICs.

### Effect of antibiotics on biofilm formation of *A*. *baumannii* clinical isolates

We analyzed the number of biofilm cells in *A*. *baumannii* after 24 hours of incubation. The number of biofilm cells in 3 strains (R4, R5, and R10) was significantly higher than those in ATCC 19606, whereas the number of biofilm cells for strain R9 was significantly lower than that in ATCC 19606 (S1B Fig). In addition, the number of biofilm cells for 3 strains (R1, R3, and R7) was higher than that in ATCC 19606. Biofilm cells in strains R2, R6, and R8 showed almost the same number of cells as in strain ATCC 19606. These results indicate that some of clinical isolates of MDRA have a high ability of biofilm formation.

Next, to clarify the effect of CST, PMB, MIN, and TGC at sub-MICs on the biofilm formation of clinical isolates of MDRA, we analyzed the number of biofilm cells in the isolates and ATCC 19606 after 24 hours of incubation with these antibiotics at their sub-MICs. Representative data of the number of biofilm cells in strains ATCC 19606, R2, and R3 are shown in Fig 1 and that of others are shown in S2 and S3 Figs. The number of biofilm cells for strains ATCC 19606, R7, R9, and R10 significantly decreased in the presence of CST in a dose-dependent manner (Fig 1A) (S3A, S3I and S3M Fig). In contrast, the number of biofilm cells for strains, R2, R3, R1, R4, and R5, increased in the presence of CST at sub-MICs (Fig 1E and 1I) (S2A, S2E and S2I Fig). In addition, the number of biofilm cells for strains, R6 and R8, were almost the same in the presence of CST as compared to that in the absence of CST (S2M and S3E Figs). The number of biofilm cells for strains, ATCC 19606, R9, and R10, significantly decreased in the presence of PMB in a dose-dependent manner (Fig 1B) (S3J and S3N Fig), as well as the number of cells for strains, R3, R4, R5, R6, R7, and R8, decreased in the presence of PMB at 1/2 MIC (Fig 1J) (S2F, S2J, S2N, S3B and S3F Figs). In contrast, the number of biofilm cells for strain R2 significantly increased in the presence of PMB at 1/2 MIC (Fig 1F). In addition, the number of biofilm cells for strain R1 were almost the same in the presence of PMB as compared to that in the absence of PMB (S2B Fig). The number of biofilm cells for strain ATCC 19606 and clinical isolates, excluding R3, significantly decreased in the presence of MIN in a dose-dependent manner (Fig 1C and 1G) (S2C, S2G, S2K, S2O, S3C, S3G, S3K and S3O Figs). In addition, the number of biofilm cells for strain R3 significantly decreased in the presence of MIN at 1/2 MIC (Fig 1K). The number of biofilm cells for strains, ATCC 19606, R3, R4, R6, R8, and R9, decreased in the presence of TGC in a dose-dependent manner (Fig 1D and 1L) (S2H, S2P, S3H and S3L Figs), as well as those in strains R2, R1, R5, R7, and R10 were significantly decreased in the presence of TGC at 1/2 MIC (Fig 1H) (S2D, S2L, S3D and S3P Figs). These results indicate that the biofilm formation of clinical isolates of MDRA is induced by CST and PMB at sub-MICs.

### Expression levels of genes encoding efflux pumps and biofilm-related proteins in the presence of CST

**Fig 2:**
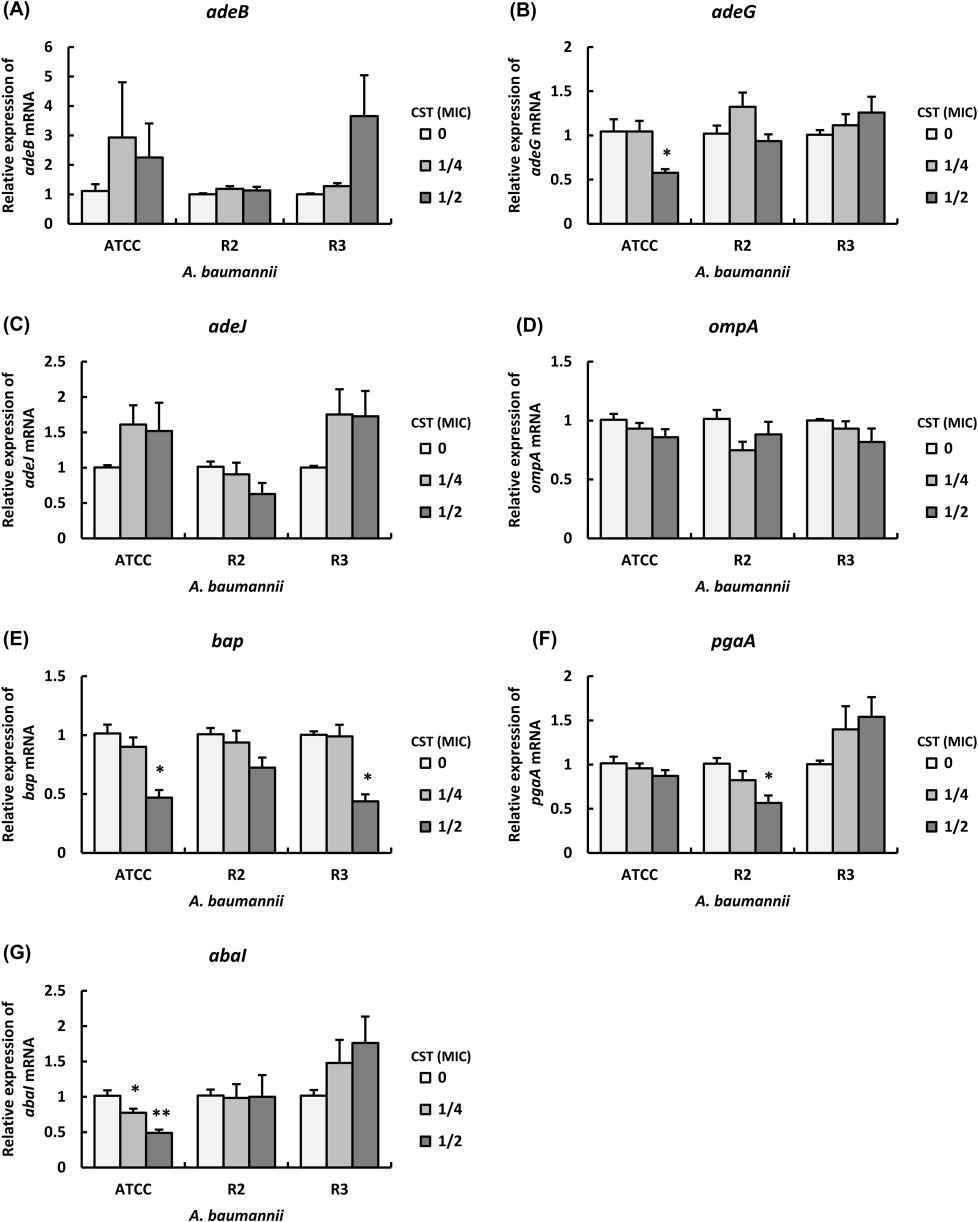
Effect of CST at sub-MICs on the expression levels of efflux pumps and biofilm-related genes in *A*. *baumannii*. Summarized results showing the mRNA levels of efflux pumps and biofilm-related genes in strains ATCC 19606, R2 and R3 cultured in LB-broth with CST at sub-MICs. The mRNA levels of (A) *adeB*, (B) *adeG*, (C) *adeJ*, (D) *ompA*, (E) *bap*, (F) *pgaA* and (G) *abaI* were analyzed by real-time PCR. Bar graph data are shown as the means ± SEM (n = 6) of 3 independent experiments. Asterisks indicate statistically significant differences (**P<0.01; *P<0.05, non-treated bacteria *vs*. antibiotics-treated bacteria; One-way ANOVA).

The biofilm formation of clinical isolates of MDRA was increased in the presence of CST and PMB at sub-MICs, as shown in Fig 1, and S2 and S3 Figs. Therefore, we analyzed whether the expression levels of genes encoding efflux pumps and biofilm-related proteins in representative strains ATCC 19606, R2, and R3 were varied by the antibiotics at sub-MICs. The mRNA levels of *adeB* in strain ATCC 19606 in the presence of CST at 1/4 and 1/2 MICs were increased compared with that in the absence of CST, as well as that in strain R3 in the presence of CST at 1/2 MIC was increased compared with that in the absence of CST (Fig 2A). On the other hand, there were no effects of CST at sub-MICs on the mRNA levels of *adeB* in strain R2 (Fig 2A). The mRNA level of *adeG* in strain ATCC 19606 in the presence of CST at 1/2 MIC was significantly decreased compared with that in the absence of CST, whereas that in strain R2 in the presence of CST at 1/4 MIC was increased compared with that in the absence of CST (Fig 2B). The mRNA levels of *adeG* in strain R3 were slightly increased by CST in a dose-dependent manner (Fig 2B). The mRNA levels of *adeJ* in strains ATCC 19606 and R3 in the presence of CST at 1/4 and 1/2 MICs were increased compared with those in the absence of CST, whereas those in strain R2 were decreased by CST in a dose-dependent manner (Fig 2C). The mRNA levels of *ompA* in strains ATCC 19606 and R3 were slightly decreased by CST in a dose-dependent manner, as well as that in strain R2 in the presence of CST at 1/4 MIC was decreased compared with that in the absence of CST (Fig 2D). The mRNA levels of *bap* in strains ATCC 19606 and R3 in the presence of CST at 1/2 MIC were significantly decreased compared with that in the absence of CST, whereas that in strain R2 in the presence of CST at 1/2 MIC was decreased compared with that in the absence of CST (Fig 2E). The mRNA levels of *pgaA* in strain R2 were decreased by CST in a dose-dependent manner, whereas those in strain R3 were increased by CST in a dose-dependent manner (Fig 2F). On the other hand, there were no effects of CST at sub-MICs on the mRNA levels of *pgaA* in strain ATCC 19606 (Fig 2F). The mRNA levels of *abaI* in strain ATCC 19606 were significantly decreased by CST in a dose-dependent manner, whereas those in strain R3 were increased by CST in a dose-dependent manner (Fig 2G). On the other hand, there were no effects of CST at sub-MICs on the mRNA levels of *abaI* in strain R2 (Fig 2G). These results indicate that CST alters the expression levels of genes encoding efflux pumps and biofilm-related proteins in *A*. *baumannii*.

### Expression levels of genes encoding efflux pumps and biofilm-related proteins in the presence of PMB

**Fig 3:**
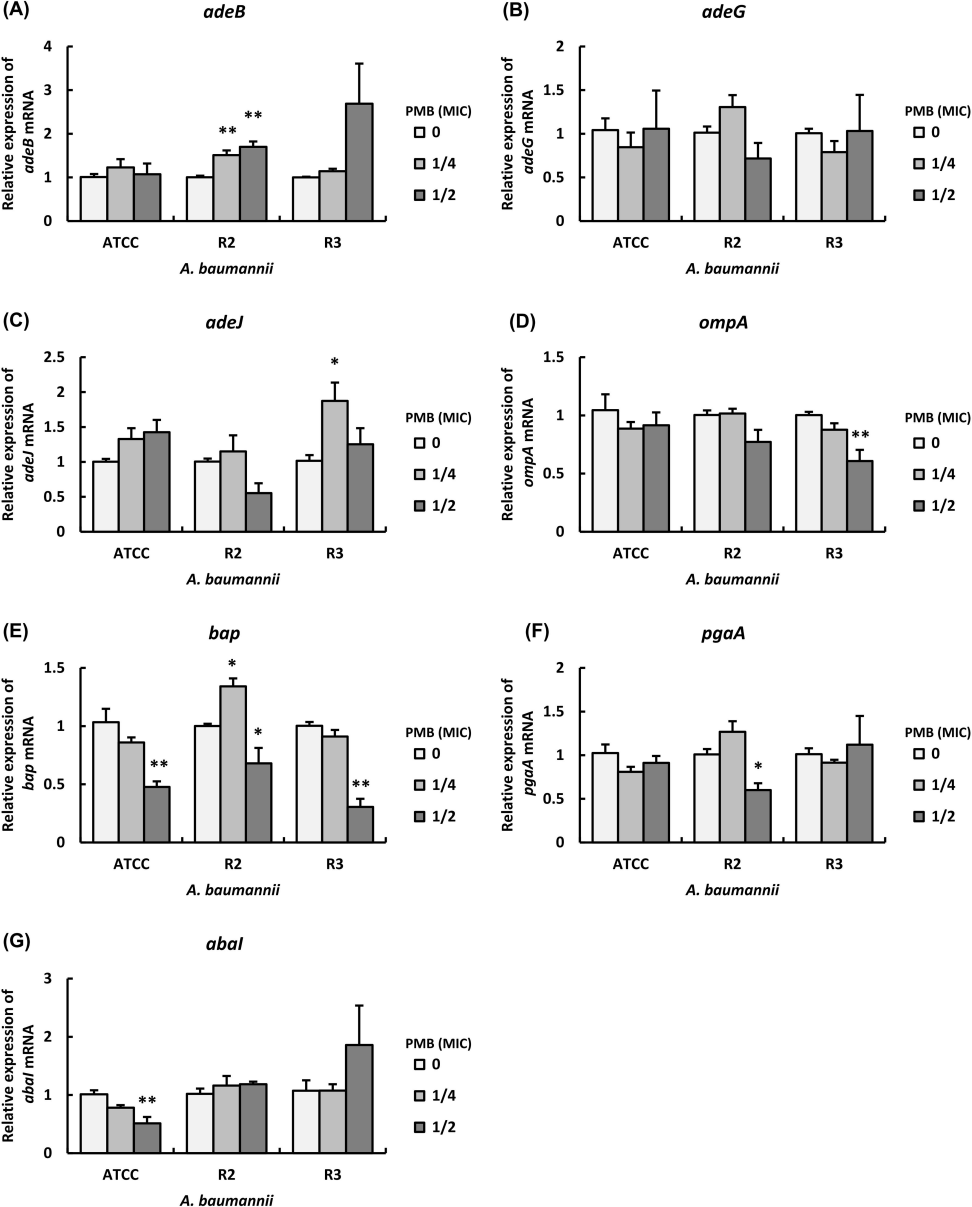
Effect of PMB at sub-MICs on the expression levels of efflux pumps and biofilm-related genes in *A*. *baumannii*. Summarized results showing the mRNA levels of efflux pumps and biofilm-related genes in strains ATCC 19606, R2 and R3 cultured in LB-broth with sub-MICs of PMB. The mRNA levels of (A) *adeB*, (B) *adeG*, (C) *adeJ*, (D) *ompA*, (E) *bap*, (F) *pgaA* and (G) *abaI* were analyzed by real-time PCR. Bar graph data are shown as the means ± SEM (n = 6) of 3 independent experiments. Asterisks indicate statistically significant differences (**P< 0.01; *P<0.05, non-treated bacteria *vs*. antibiotics-treated bacteria; One-way ANOVA).

Next, we analyzed the expression levels of genes encoding efflux pumps and biofilm-related genes in representative strains ATCC 19606, R2, and R3 in the presence of PMB at sub-MICs. The mRNA levels of *adeB* in strain R2 were significantly increased by PMB in a dose-dependent manner, as well as that in strain R3 in the presence of PMB at 1/2 MIC was increased compared with that in the absence of PMB (Fig 3A). On the other hand, there were no effects of PMB at sub-MICs on the mRNA levels of *adeB* in strain ATCC 19606 (Fig 3A). The mRNA levels of *adeG* in strains ATCC 19606 and R3 in the presence of PMB at 1/4 MIC were decreased compared with those in the absence of PMB (Fig 3B). On the other hand, the mRNA levels of *adeG* in strain R2 in the presence of PMB at 1/4 and 1/2 MICs were increased and decreased respectively, compared with that in the absence of PMB (Fig 3B). The mRNA levels of *adeJ* in strain ATCC 19606 were increased by PMB in a dose-dependent manner, as well as that in strain R3 in the presence of PMB at 1/4 MIC was significantly increased compared with that in the absence of PMB (Fig 3C). On the other hand, the mRNA level of *adeJ* in strain R2 in the presence of PMB at 1/2 MIC was decreased compared with that in the absence of PMB (Fig 3C). The mRNA levels of *ompA* in strain R3 were significantly decreased by PMB in a dose-dependent manner, as well as that in strain R2 in the presence of PMB at 1/2 MIC was decreased compared with that in the absence of PMB (Fig 3D). In addition, the mRNA levels of *ompA* in strain ATCC 19606 in the presence of PMB at 1/4 and 1/2 MICs were slightly decreased compared with that in the absence of PMB (Fig 3D). The mRNA levels of *bap* in strains ATCC 19606 and R3 were significantly decreased by PMB in a dose-dependent manner, whereas those in strain R2 in the presence of PMB at 1/4 and 1/2 MICs were increased and decreased respectively, compared with that in the absence of PMB (Fig 3E). The mRNA levels of *pgaA* in strain ATCC 19606 in the presence of PMB at 1/4 and 1/2 MICs were slightly decreased compared with that in the absence of PMB, whereas those in strain R3 in the presence of PMB at 1/4 and 1/2 MICs were slightly decreased and increased respectively, compared with that in the absence of PMB (Fig 3F). On the other hand, the mRNA levels of *pgaA* in strain R2 in the presence of PMB at 1/4 and 1/2 MICs were increased and decreased respectively, compared with that in the absence of PMB (Fig 3F). The mRNA levels of *abaI* in strain ATCC 19606 were significantly decreased by CST in a dose-dependent manner, whereas that in strain R3 in the presence of PMB at 1/2 MIC was increased compared with that in the absence of PMB (Fig 3G). In addition, there were no effects of PMB at sub-MICs on the mRNA levels of *abaI* in strain R2 (Fig 3G). These results indicate that PMB alters the expression levels of genes encoding efflux pumps and biofilm-related proteins in *A*. *baumannii*.

### Correlation between biofilm formation and expression levels of genes encoding efflux pumps and biofilm-related proteins in the presence of CST and PMB

Although we clarified that the treatment with CST and PMB at their sub-MICs alters the expression levels of genes encoding efflux pumps and biofilm-related proteins in *A*. *baumannii*, the specific genes that are directly associated with the biofilm formation of *A*. *baumannii* in the presence of CST and PMB at their sub-MICs remain unclear. Therefore, we calculated the correlation of biofilm formation with the mRNA levels of efflux pumps and biofilm-related genes in *A*. *baumannii* after incubation with CST and PMB at sub-MICs. Pearson correlation analysis revealed a positive and significant correlation between the number of biofilm cells and the mRNA levels of *adeG*, *ompA*, *bap*, *pgaA*, and *abaI* in strain ATCC 19606 in the presence of CST (Table 3 and S4 Fig). In addition, Pearson correlation analysis revealed a positive and significant correlation between the number of biofilm cells and the mRNA levels of *adeB*, *adeG*, and *abaI* in strain R2 in the presence of CST as well (Table 3 and S5 Fig). It further revealed a positive and significant correlation between the number of biofilm cells and the mRNA levels of *adeB*, *adeG*, *adeJ*, *pgaA*, and *abaI* in strain R3 in the presence of CST (Table 3 and S6 Fig). Furthermore, Pearson correlation analysis revealed a positive and significant correlation between the number of biofilm cells and the mRNA levels of *bap* and *abaI* in strain ATCC 19606 in the presence of PMB (Table 4 and S7 Fig). In addition, Pearson correlation analysis revealed a positive and significant correlation between the number of biofilm cells and the mRNA levels of *adeB* in strain R2 in the presence of PMB (Table 4 and S8 Fig). A positive and significant correlation was also observed between the number of biofilm cells and the mRNA levels of *ompA* and *bap* in strain R3 in the presence of PMB (Table 4 and S9 Fig). These results suggest that CST and PMB alter the biofilm formation of *A*. *baumannii* depending on the regulation of their efflux pumps and biofilm-related gene expression.

**Table 3:** Relationship between biofilm formation and the expression of efflux pumps and biofilm-related genes in *A*. *baumannii* in the presence of CST at its sub-MICs.

*A*. *baumannii*		Factor	*adeB*	*adeG*	*adeJ*	*ompA*	*bap*	*pgaA*	*abaI*
ATCC 19606		Biofilm formation	0.096	0.821**	−0.023	0.597**	0.935**	0.571*	0.905**
MDRA	R2		0.922**	0.624**	0.425	0.459	0.448	0.242	0.787**
	R3		0.726**	0.797**	0.842**	0.362	−0.060	0.855**	0.882**

Pearson correlation coefficients were calculated for ATCC19606, R2, and R3 strains cultured with different sub-MIC concentrations (0, 1/4, and 1/2 the MIC) of CST.

** Significant at the level of 0.01 (2-tailed).

* Significant at the level of 0.05 (2-tailed).

**Table 4:** Relationship between biofilm formation and the expression of efflux pumps and biofilm-related genes in *A*. *baumannii* in the presence of PMB at its sub-MICs.

*A*. *baumannii*		Factor	*adeB*	*adeG*	*adeJ*	*ompA*	*bap*	*pgaA*	*abaI*
ATCC 19606		Biofilm formation	0.196	0.212	−0.344	0.169	0.919**	0.058	0.743**
MDRA	R2		0.754**	0.131	0.046	−0.027	−0.087	−0.155	0.464
	R3		−0.121	0.418	0.450	0.948**	0.953**	0.344	0.138

Pearson correlation coefficients were calculated for ATCC19606, R2, and R3 strains cultured with different sub-MIC concentrations (0, 1/4, and 1/2 the MIC) of PMB.

** Significant at the level of 0.01 (2-tailed).

## Discussion

*A*. *baumannii* has recently emerged as a major nosocomial pathogen [1–3] and an increase in the outbreaks of MDRA worldwide is becoming a cause for concern [8, 9]. Although previous studies have clarified the mechanisms of antibiotic resistance in *A*. *baumannii*, there are relatively few research studies that investigate the effect of antibiotics at the sub-MICs on the pathogenicity of *A*. *baumannii*. Therefore, we aimed to elaborate the effect of antibiotics, commonly used for the treatment of MDRA infections, on the growth and biofilm formation of clinical isolates of MDRA.

Polymyxins are membrane-active peptides with bactericidal capabilities of disorganizing of the outer bacterial cell-membrane [21], they are currently used as a last-resort antibiotic for the treatment of multidrug-resistant gram-negative bacterial infections [22]. Our study demonstrated that the number of planktonic cells in strain ATCC 19606 decreased in the presence of CST and PMB at their sub-MICs in a dose-dependent manner. However, the number of planktonic cells in five and one out of every ten clinical isolates of MDRA was slightly decreased in the presence of CST and PMB, respectively. This indicates that a part of MDRA continues to grow below the MICs of these antibiotics. Antibiotic concentrations are often below the MIC in cellular compartments and tissues [16]. Therefore, exposure to a lethal dose i.e., bactericidal concentration, of polymyxins in patients is important for the treatment of *A*. *baumannii* infection, although polymyxins often exhibit adverse side effects, such as nephro- and neurotoxicity, and are thus not suitable for treating all types of infections [29].

Previous studies have reported that CST considerably decreases biofilm formation of clinical isolates of *A*. *baumannii* and *Klebsiella pneumoniae* at its sub-MIC [22, 30]. However, the CST at its sub-MICs is reported to induce the *Pseudomonas* quinolone signal in *Pseudomonas aeruginosa*, indicating the upregulation of biofilm formation [31]. We observed that the number of biofilm cells in 7 clinical isolates of MDRA was increased and not altered by CST at sub-MICs. These results suggest that CST often increases biofilm formation of MDRA, resulting bacterial biofilm infections i.e. the protection from killing by antibiotics, and the evasion or tolerance of host immune responses [32]. On the other hand, we observed that the number of biofilm cells in 3 clinical isolates of MDRA was increased or not altered by PMB at its sub-MICs. A previous study reported that PMB had no induction of biofilm formation in *P*. *aeruginosa* [33]. These results suggest that PMB rather than CST shows few effects on the induction of biofilm formation of gram-negative bacteria. However, exposure to a lethal dose of bactericidal concentration of polymyxins in patients is necessary for the treatment of *A*. *baumannii* infection to be effective in killing bacteria.

We suggest that the biofilm formation in strain ATCC 19606 and representative 2 clinical isolates of MDRA (R2 and R3) are correlated with AdeFGH efflux pump and autoinducer synthase AbaI in the presence of CST at sub-MICs, as well as those of the clinical isolates are also correlated with AdeABC in them. Although three RND systems, AdeABC, AdeFGH, and AdeIJK have been associated with multidrug resistance in *A*. *baumannii* [5], there were few reports regarding the role of these pumps in biofilm formation. He et al. demonstrated that the biofilm formation of *A*. *baumannii* clinical isolates was associated with overexpression of AdeFGH and AbaI in the presence of LVX and MEM at sub-MICs and discussed that the overexpression of AdeFGH accelerated the synthesis and transport of AHLs during biofilm formation in *A*. *baumannii* [25]. These results, in addition to those of our study, suggest that various antibiotics at sub-MICs have potential effects on the development of biofilms in *A*. *baumannii* via the combined upregulation of AdeFGH and AbaI. In addition, our results suggest that the expression of AdeABC in clinical isolates of MDRA is also correlated with the biofilm formation in the presence of CST at sub-MICs, although AdeABC contributes to the resistance of carbapenem [34]. However, the gene expression of *adeABC* is tightly regulated by the two-component regulatory system AdeR-AdeS [35], whereas that of *adeFGH* is regulated by a putative LysR-type transcriptional regulator (LTTR), named *adeL* [36]. However, the mechanisms underlying their gene expressions is different; therefore, further studies are required to determine the induction mechanisms of these genes in the presence of antibiotics.

Biofilm formation is positively correlated with the expression of PNAG and Bap [26]. The mRNA levels of *pgaA* in strains ATCC 19606 and R3 were correlated with the biofilm formation in the presence of CST at sub-MICs, as well as those of *bap* in those strains were also correlated with the biofilm formation in the presence of PMB at sub-MICs, suggesting that CST and PMB alter biofilm formation via the regulation of PNAG and Bap. PNAG and Bap play important roles in colonization, adherence, and immune evasion in infections as well as biofilm formation [11, 26], so that the virulence of *A*. *baumannii* clinical isolates appears to be altered in the presence of CST and PMB at sub-MICs. Furthermore, the expression levels of *adeJ* and *ompA* in strain R3 were correlated with biofilm formation in the presence of CST and PMB at sub-MICs, respectively. Both AdeIJK and OmpA transport the virulence factors in *A*. *baumannii* [10, 26], so that the virulence, including biofilm formation of some clinical isolates of MDRA, appears to be altered in the presence of CST and PMB at sub-MICs.

MIN is a semisynthetic derivative of tetracycline and its derivative TGC is effective for the treatment of MDRA [2, 5]. Our study demonstrated that the number of planktonic and biofilm cells in clinical isolates of MDRA were significantly decreased by MIN and TGC at sub-MICs in a dose-dependent manner in contrast to the treatment of CST and PMB at sub-MICs, indicating that both bacterial growth and biofilm formation are inhibited in the presence of MIN and TGC at sub-MICs. Previous studies suggest that a combination of TGC with CST, LVX, amikacin, and IPM may be an effective therapy to synergistically prevent the emergence of resistance during treatment of MDRA infections [37, 38]. Presumably, although the treatment of polymyxins or LVX alone can induce biofilm formation and confer bacteria with resistance against antibiotics ([25], in this study), the combination of TGC with polymyxins or LVX shows more favorable therapeutic outcomes against MDRA infections.

In summary, we demonstrated that the renewed biofilm formation of clinical isolates of MDRA depends on polymyxins at the sub-MICs. Biofilm formation promotes the progress of *A*. *baumannii* infection as shown in previous studies and consequently, blocking efflux pumps and biofilm-related proteins may contribute to their treatment. Further studies are required to understand the mechanism of biofilm formation associated with the treatment of antibiotics in *A*. *baumannii* infection.

## Supporting information

S1 Table MICs (μg/mL) of antibiotics against the clinical isolates of MDRA. (DOCX)

S1 Fig Number of planktonic and biofilm cells in ATCC 19606 and clinical isolates of MDRA. Summarized results showing the number of (A) planktonic and (B) biofilm cells in strains ATCC 19606 and clinical isolates of MDRA cultured in LB broth for 24 hours at 37°C. After the culture, the supernatant from each well was transferred to the corresponding well in a new plate and the OD was measured at 595 nm; the number of planktonic cells was defined. The biofilm cells were stained with 1% crystal violet solution for 15 minutes, as shown in the Methods. The stained biofilm cells were de-stained with 95% ethanol and the OD was measured at 595 nm; the number of biofilm cells was defined. Asterisks indicate statistically significant differences (**P<0.01; *P<0.05, ATCC 19606 *vs*. clinical isolate; Student’s *t*-test).
(TIF)

S2 Fig Effect of four antibiotics at sub-MICs on bacterial growth and biofilm formation of *A. baumannii*. Summarized results showing the ratio of planktonic and biofilm cells in strain R1 cultured in LB broth with (A) CST, (B) PMB, (C) MIN and (D) TGC at sub-MICs. Summarized results showing the ratio of planktonic and biofilm cells in strain R4 cultured in LB broth with (E) CST, (F) PMB, (G) MIN and (H) TGC at sub-MICs. Summarized results showing the ratio of planktonic and biofilm cells in strain R5 cultured in LB broth with (I) CST, (J) PMB, (K) MIN and (L) TGC at sub-MICs. Summarized results showing the ratio of planktonic and biofilm cells in strain R6 cultured in LB broth with (M) CST, (N) PMB, (O) MIN and (P) TGC at sub-MICs. Dark gray and gray bars indicate the ratio of planktonic and biofilm cells in *A*. *baumannii*, respectively. Bar graph data are shown as the mean ± SEM (n = 6) of 3 independent experiments. Asterisks indicate statistically significant differences in the number of planktonic cells (**P<0.01; *P<0.05, non-treated bacteria *vs*. antibiotics-treated bacteria; One-way ANOVA). Crosses indicate statistically significant differences in the number of biofilm cells (^††^*P*<0.01; ^†^*P*<0.05, non-treated bacteria *vs*. antibiotics-treated bacteria; One-way ANOVA).
(TIF)

S3 Fig Effect of four antibiotics at sub-MICs on bacterial growth and biofilm formation of *A. baumannii*. Summarized results showing the ratio of planktonic and biofilm cells in strain R7 cultured in LB broth with (A) CST, (B) PMB, (C) MIN and (D) TGC at sub-MICs. Summarized results showing the ratio of planktonic and biofilm cells in strain R8 cultured in LB broth with (E) CST, (F) PMB, (G) MIN and (H) TGC at sub-MICs. Summarized results showing the ratio of planktonic and biofilm cells in strain R9 cultured in LB broth with (I) CST, (J) PMB, (K) MIN and (L) TGC at sub-MICs. Summarized results showing the ratio of planktonic and biofilm cells in strain R10 cultured in LB broth with (M) CST, (N) PMB, (O) MIN and (P) TGC at sub-MICs. Dark gray and gray bars indicate the ratio of planktonic and biofilm cells in *A*. *baumannii*, respectively. Bar graph data are shown as the mean ± SEM (n = 6) of 3 independent experiments. Asterisks indicate statistically significant differences in the number of planktonic cells (**P<0.01; *P<0.05, non-treated bacteria *vs*. antibiotics-treated bacteria; One-way ANOVA). Crosses indicate statistically significant differences in the number of biofilm cells (^††^*P*<0.01; ^†^*P*<0.05, non-treated bacteria *vs*. antibiotics-treated bacteria; One-way ANOVA).
(TIF)

S4 Fig Relationship between the biofilm formation and gene expression in strain ATCC 19606 in the presence of CST at sub-MICs. Pearson correlation coefficient was calculated for the number of biofilm cells and the expression of efflux pumps and biofilm-related genes in strain ATCC 19606. (A) *adeB* mRNA (Pearson correlation coefficient r = 0.096, *P* = 0.706), (B) *adeG* mRNA (Pearson correlation coefficient r = 0.821, *P*<0.001), (C) *adeJ* mRNA (Pearson correlation coefficient r = −0.023, *P* = 0.928), (D) *ompA* mRNA (Pearson correlation coefficient r = 0.597, *P* = 0.009), (E) *bap* mRNA (Pearson correlation coefficient r = 0.935, *P*<0.001), (F) *pgaA* mRNA (Pearson correlation coefficient r = 0.571, *P* = 0.013), and (G) *abaI* mRNA (Pearson correlation coefficient r = 0.905, *P*<0.001). Each symbol represents ATCC19606 strain in the absence and presence of CST.
(TIF)

S5 Fig Relationship between the biofilm formation and gene expression in strain R2 in the presence of CST at sub-MICs. Pearson correlation coefficient was calculated for the number of biofilm cells and the expression of efflux pumps and biofilm-related genes in strain R2. (A) *adeB* mRNA (Pearson correlation coefficient r = 0.922, *P*<0.001), (B) *adeG* mRNA (Pearson correlation coefficient r = 0.624, *P* = 0.006), (C) *adeJ* mRNA (Pearson correlation coefficient r = 0.425, *P* = 0.079), (D) *ompA* mRNA (Pearson correlation coefficient r = 0.459, *P* = 0.056), (E) *bap* mRNA (Pearson correlation coefficient r = 0.448, *P* = 0.062), (F) *pgaA* mRNA (Pearson correlation coefficient r = 0.242, *P* = 0.332), and (G) *abaI* mRNA (Pearson correlation coefficient r = 0.787, *P*<0.001). Each symbol represents R2 strain in the absence and presence of CST.
(TIF)

S6 Fig Relationship between the biofilm formation and gene expression in strain R3 in the presence of CST at sub-MICs. Pearson correlation coefficient was calculated for the number of biofilm cells and the expression of efflux pumps and biofilm-related genes in strain R3. (A) *adeB* mRNA (Pearson correlation coefficient r = 0.726, *P*<0.001), (B) *adeG* mRNA (Pearson correlation coefficient r = 0.797, *P*<0.001), (C) *adeJ* mRNA (Pearson correlation coefficient r = 0.842, *P*<0.001), (D) *ompA* mRNA (Pearson correlation coefficient r = 0.362, *P* = 0.139), (E) *bap* mRNA (Pearson correlation coefficient r = −0.060, *P* = 0.812), (F) *pgaA* mRNA (Pearson correlation coefficient r = 0.855, *P*<0.001), and (G) *abaI* mRNA (Pearson correlation coefficient r = 0.882, *P*<0.001). Each symbol represents R3 strain in the absence and presence of CST.
(TIF)

S7 Fig Relationship between the biofilm formation and gene expression in strain ATCC 19606 in the presence of PMB at sub-MICs. Pearson correlation coefficient was calculated for the number of biofilm cells and the expression of efflux pumps and biofilm-related genes in strain ATCC 19606. (A) *adeB* mRNA (Pearson correlation coefficient r = 0.196, *P* = 0.435), (B) *adeG* mRNA (Pearson correlation coefficient r = 0.212, *P* = 0.398), (C) *adeJ* mRNA (Pearson correlation coefficient r = −0.344, *P* = 0.162), (D) *ompA* mRNA (Pearson correlation coefficient r = 0.169, *P* = 0.502), (E) *bap* mRNA (Pearson correlation coefficient r = 0.919, *P*<0.001), (F) *pgaA* mRNA (Pearson correlation coefficient r = 0.058, *P* = 0.820), and (G) *abaI* mRNA (Pearson correlation coefficient r = 0.743, *P*<0.001). Each symbol represents ATCC19606 strain in the absence and presence of PMB.
(TIF)

S8 Fig Relationship between the biofilm formation and gene expression in strain R2 in the presence of PMB at sub-MICs. Pearson correlation coefficient was calculated for the number of biofilm cells and the expression of efflux pumps and biofilm-related genes in strain R2. (A) *adeB* mRNA (Pearson correlation coefficient r = 0.754, *P*<0.001), (B) *adeG* mRNA (Pearson correlation coefficient r = 0.131, *P* = 0.604), (C) *adeJ* mRNA (Pearson correlation coefficient r = 0.046, *P* = 0.857), (D) *ompA* mRNA (Pearson correlation coefficient r = −0.027, *P* = 0.914), (E) *bap* mRNA (Pearson correlation coefficient r = −0.087, *P* = 0.733), (F) *pgaA* mRNA (Pearson correlation coefficient r = −0.155, *P* = 0.539), and (G) *abaI* mRNA (Pearson correlation coefficient r = 0.464, *P* = 0.053). Each symbol represents R2 strain in the absence and presence of PMB.
(TIF)

S9 Fig Relationship between the biofilm formation and gene expression in strain R3 in the presence of PMB at sub-MICs. Pearson correlation coefficient was calculated for the number of biofilm cells and the expression of efflux pumps and biofilm-related genes in strain R3. (A) *adeB* mRNA (Pearson correlation coefficient r = −0.121, *P* = 0.631), (B) *adeG* mRNA (Pearson correlation coefficient r = 0.418, *P* = 0.084), (C) *adeJ* mRNA (Pearson correlation coefficient r = 0.450, *P* = 0.061), (D) *ompA* mRNA (Pearson correlation coefficient r = 0.948, *P*<0.001), (E) *bap* mRNA (Pearson correlation coefficient r = 0.953, *P*<0.001), (F) *pgaA* mRNA (Pearson correlation coefficient r = 0.344, *P* = 0.162), and (G) *abaI* mRNA (Pearson correlation coefficient r = 0.138, *P* = 0.586). Each symbol represents R3 strain in the absence and presence of PMB.
(TIF)
