# Supplementary material for: Sub-minimum inhibitory concentrations of colistin and polymyxin B promote Acinetobacter baumannii biofilm formation
Source: PLoS One. 2018 Mar 19;13(3):e0194556. doi: 10.1371/journal.pone.0194556 (PMC5858813; doi:10.1371/journal.pone.0194556)
Supplement: S1 Table — (DOCX) [file pone.0194556.s001.docx]

**S1 Table. MICs (μg/mL) of antibiotics against the clinical isolates of MDRA.**

|  | | Antibiotics | | | |
| --- | --- | --- | --- | --- | --- |
| *A. baumannii* | | Colistin  (CST) | Polymyxin B (PMB) | Minocycline (MIN) | Tigecycline (TGC) |
| MDRA | R1 | 2 | 2 | 4 | 0.5 |
|  | R4 | 2 | 2 | 4 | 0.5 |
|  | R5 | 2 | 2 | 4 | 0.5 |
|  | R6 | 2 | 2 | 4 | 1 |
|  | R7 | 4 | 2 | 4 | 0.5 |
|  | R8 | 2 | 2 | 4 | 1 |
|  | R9 | 2 | 2 | 8 | 0.5 |
|  | R10 | 2 | 2 | 0.5 | 0.5 |
