# Supplementary figures and images for: Sub-minimum inhibitory concentrations of colistin and polymyxin B promote Acinetobacter baumannii biofilm formation
Source: PLoS One. 2018 Mar 19;13(3):e0194556. doi: 10.1371/journal.pone.0194556 (PMC5858813; doi:10.1371/journal.pone.0194556)

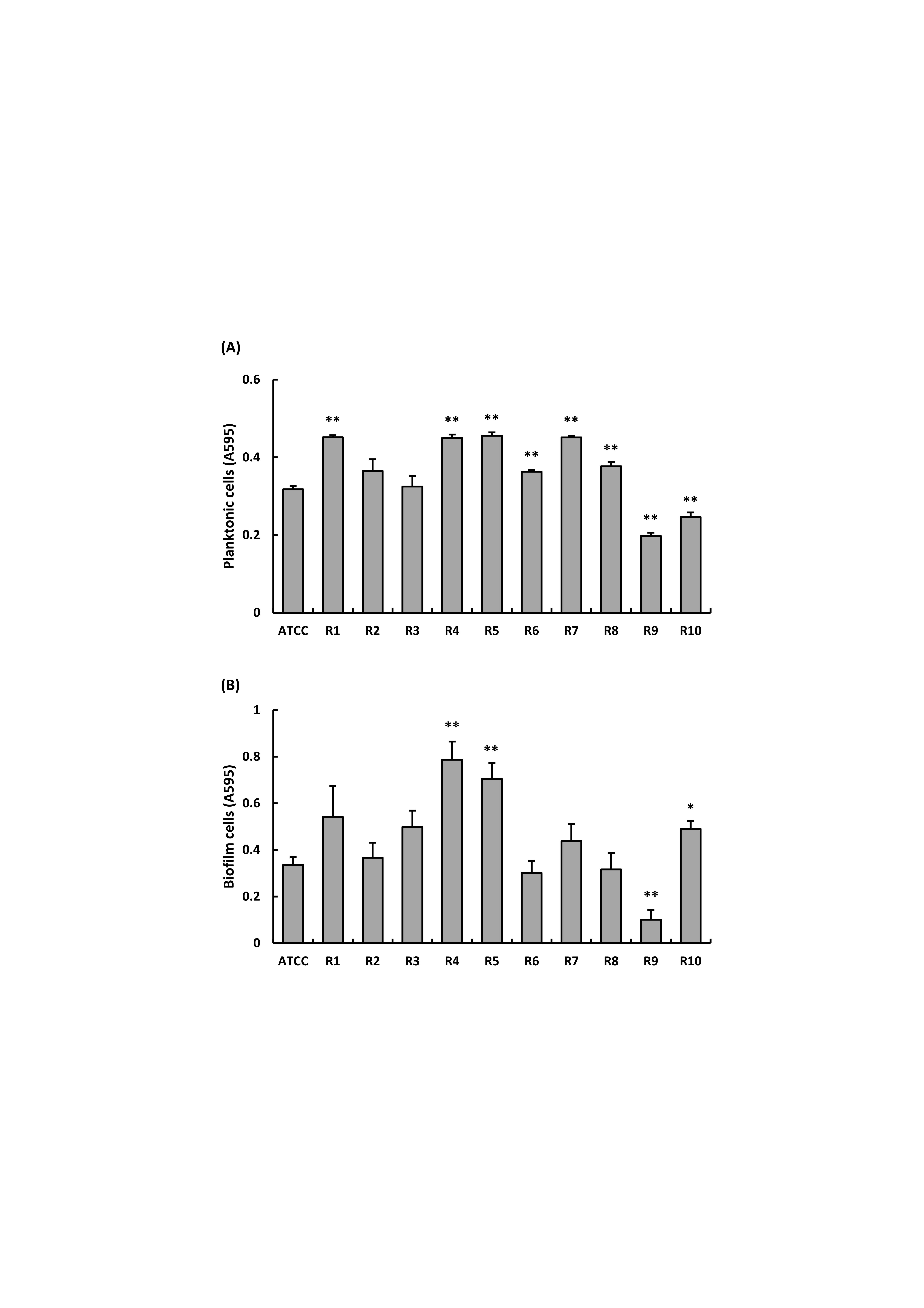

Supplement: S1 Fig — Summarized results showing the number of (A) planktonic and (B) biofilm cells in strains ATCC 19606 and clinical isolates of MDRA cultured in LB broth for 24 hours at 37°C. After the culture, the supernatant from each well was transferred to the corresponding well in a new plate and the OD was measured at 595 nm; the number of planktonic cells was defined. The biofilm cells were stained with 1% crystal violet solution for 15 minutes, as shown in the Methods. The stained biofilm cells were de-stained with 95% ethanol and the OD was measured at 595 nm; the number of biofilm cells was defined. Asterisks indicate statistically significant differences (**P<0.01; *P<0.05, ATCC 19606 vs. clinical isolate; Student’s t-test). (TIF) [file pone.0194556.s002.tif]

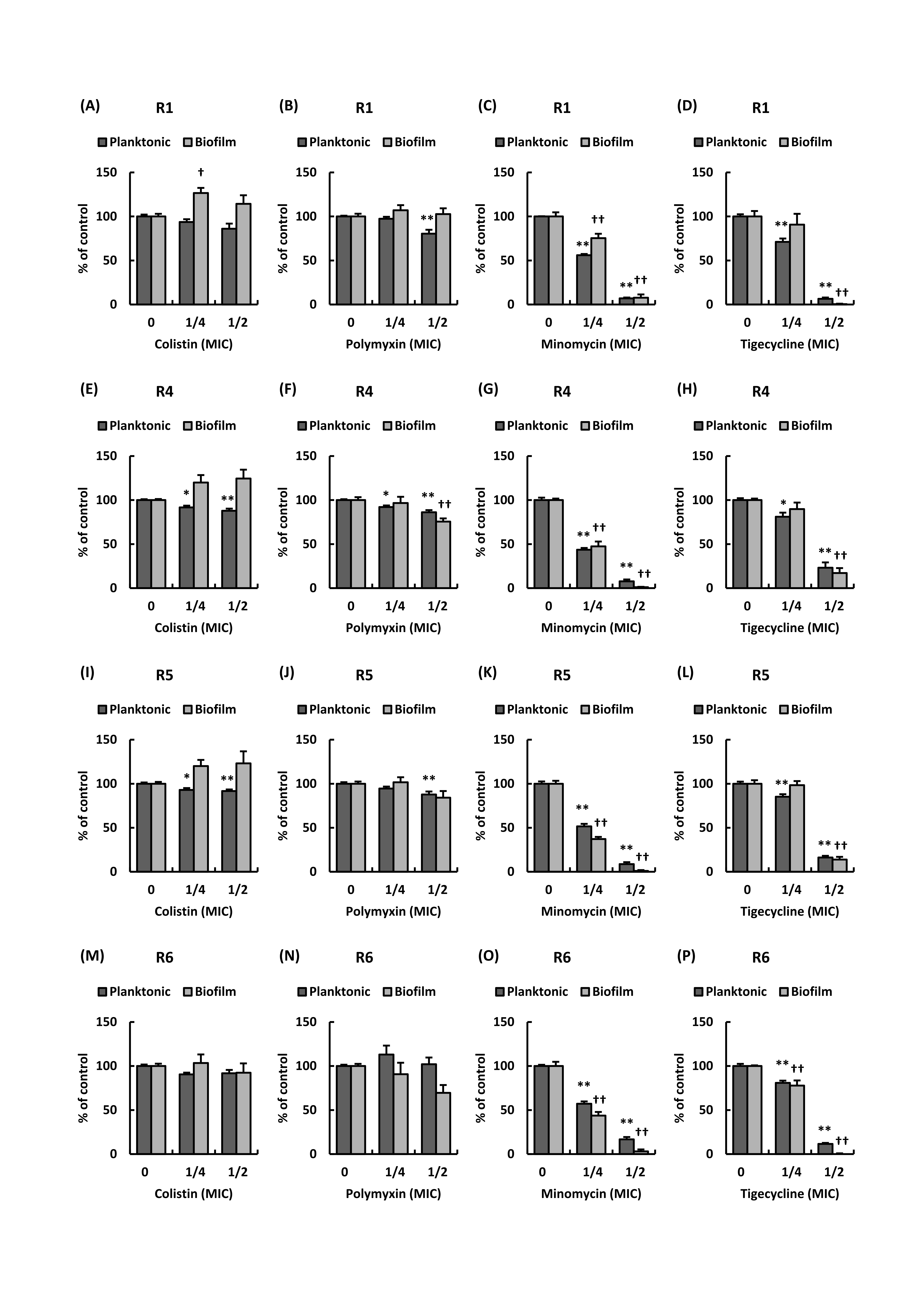

Supplement: S2 Fig — Summarized results showing the ratio of planktonic and biofilm cells in strain R1 cultured in LB broth with (A) CST, (B) PMB, (C) MIN and (D) TGC at sub-MICs. Summarized results showing the ratio of planktonic and biofilm cells in strain R4 cultured in LB broth with (E) CST, (F) PMB, (G) MIN and (H) TGC at sub-MICs. Summarized results showing the ratio of planktonic and biofilm cells in strain R5 cultured in LB broth with (I) CST, (J) PMB, (K) MIN and (L) TGC at sub-MICs. Summarized results showing the ratio of planktonic and biofilm cells in strain R6 cultured in LB broth with (M) CST, (N) PMB, (O) MIN and (P) TGC at sub-MICs. Dark gray and gray bars indicate the ratio of planktonic and biofilm cells in A. baumannii, respectively. Bar graph data are shown as the mean ± SEM (n = 6) of 3 independent experiments. Asterisks indicate statistically significant differences in the number of planktonic cells (**P<0.01; *P<0.05, non-treated bacteria vs. antibiotics-treated bacteria; One-way ANOVA). Crosses indicate statistically significant differences in the number of biofilm cells (††P<0.01; †P<0.05, non-treated bacteria vs. antibiotics-treated bacteria; One-way ANOVA). (TIF) [file pone.0194556.s003.tif]

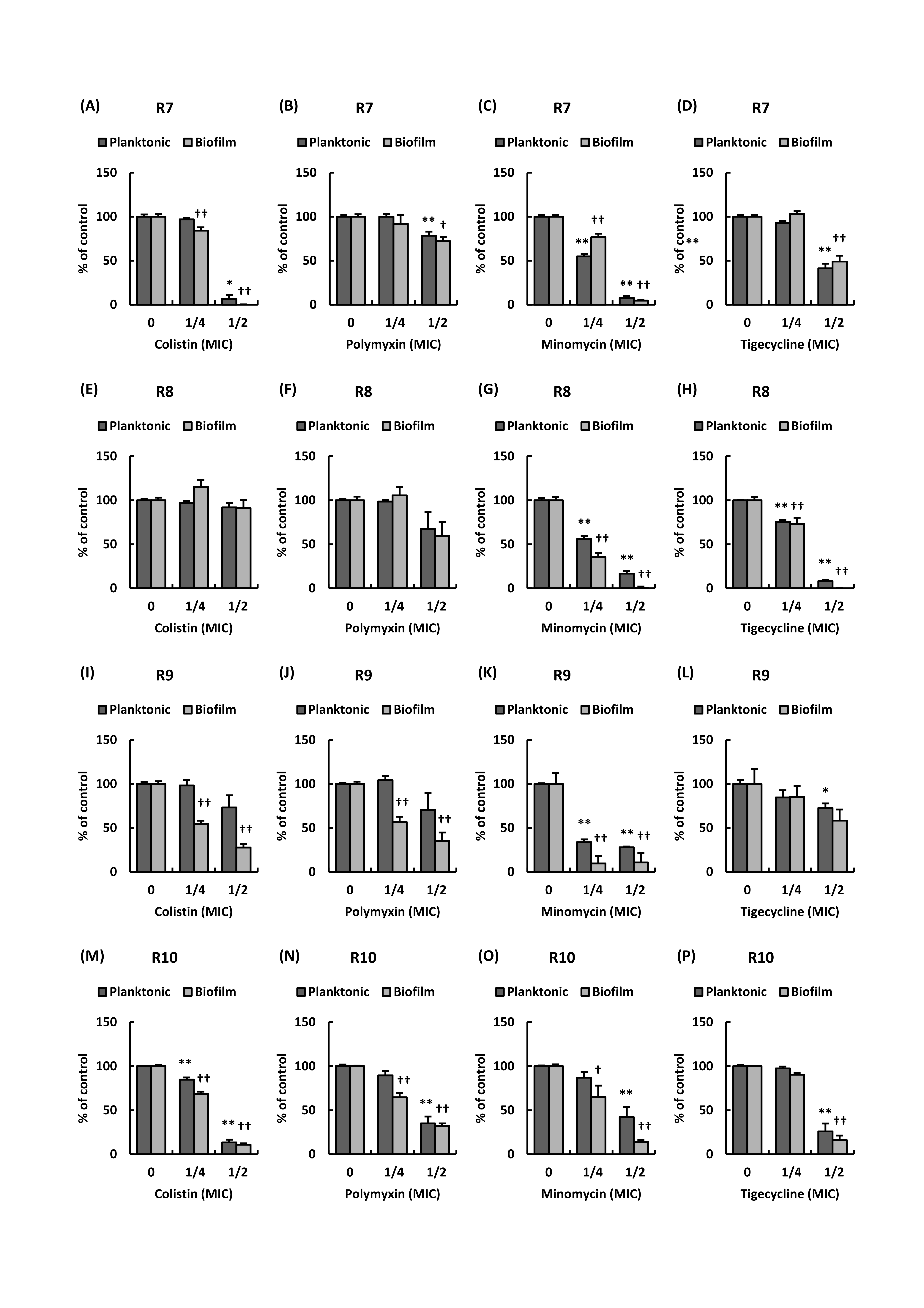

Supplement: S3 Fig — Summarized results showing the ratio of planktonic and biofilm cells in strain R7 cultured in LB broth with (A) CST, (B) PMB, (C) MIN and (D) TGC at sub-MICs. Summarized results showing the ratio of planktonic and biofilm cells in strain R8 cultured in LB broth with (E) CST, (F) PMB, (G) MIN and (H) TGC at sub-MICs. Summarized results showing the ratio of planktonic and biofilm cells in strain R9 cultured in LB broth with (I) CST, (J) PMB, (K) MIN and (L) TGC at sub-MICs. Summarized results showing the ratio of planktonic and biofilm cells in strain R10 cultured in LB broth with (M) CST, (N) PMB, (O) MIN and (P) TGC at sub-MICs. Dark gray and gray bars indicate the ratio of planktonic and biofilm cells in A. baumannii, respectively. Bar graph data are shown as the mean ± SEM (n = 6) of 3 independent experiments. Asterisks indicate statistically significant differences in the number of planktonic cells (**P<0.01; *P<0.05, non-treated bacteria vs. antibiotics-treated bacteria; One-way ANOVA). Crosses indicate statistically significant differences in the number of biofilm cells (††P<0.01; †P<0.05, non-treated bacteria vs. antibiotics-treated bacteria; One-way ANOVA). (TIF) [file pone.0194556.s004.tif]

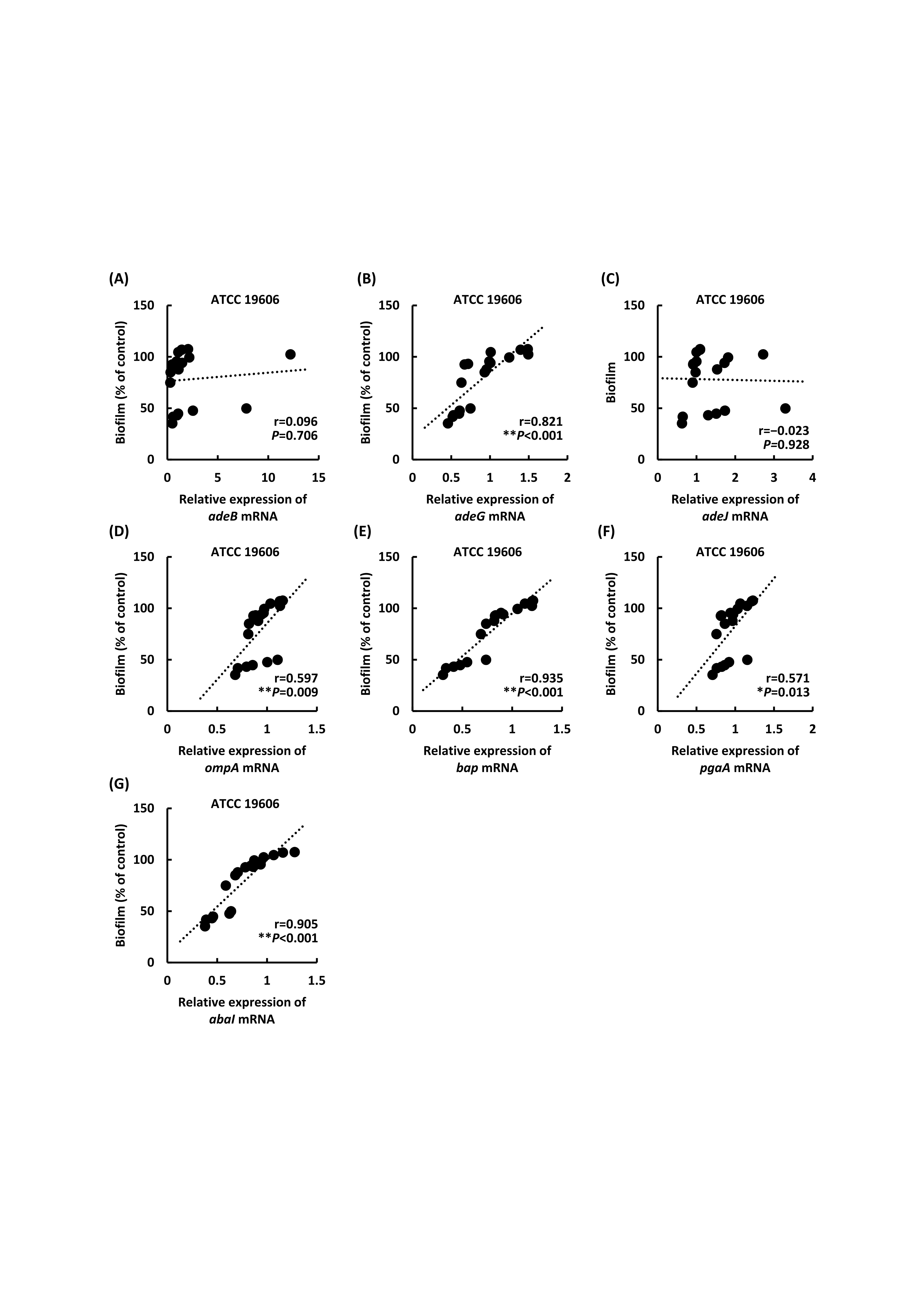

Supplement: S4 Fig — Pearson correlation coefficient was calculated for the number of biofilm cells and the expression of efflux pumps and biofilm-related genes in strain ATCC 19606. (A) adeB mRNA (Pearson correlation coefficient r = 0.096, P = 0.706), (B) adeG mRNA (Pearson correlation coefficient r = 0.821, P<0.001), (C) adeJ mRNA (Pearson correlation coefficient r = −0.023, P = 0.928), (D) ompA mRNA (Pearson correlation coefficient r = 0.597, P = 0.009), (E) bap mRNA (Pearson correlation coefficient r = 0.935, P<0.001), (F) pgaA mRNA (Pearson correlation coefficient r = 0.571, P = 0.013), and (G) abaI mRNA (Pearson correlation coefficient r = 0.905, P<0.001). Each symbol represents ATCC19606 strain in the absence and presence of CST. (TIF) [file pone.0194556.s005.tif]

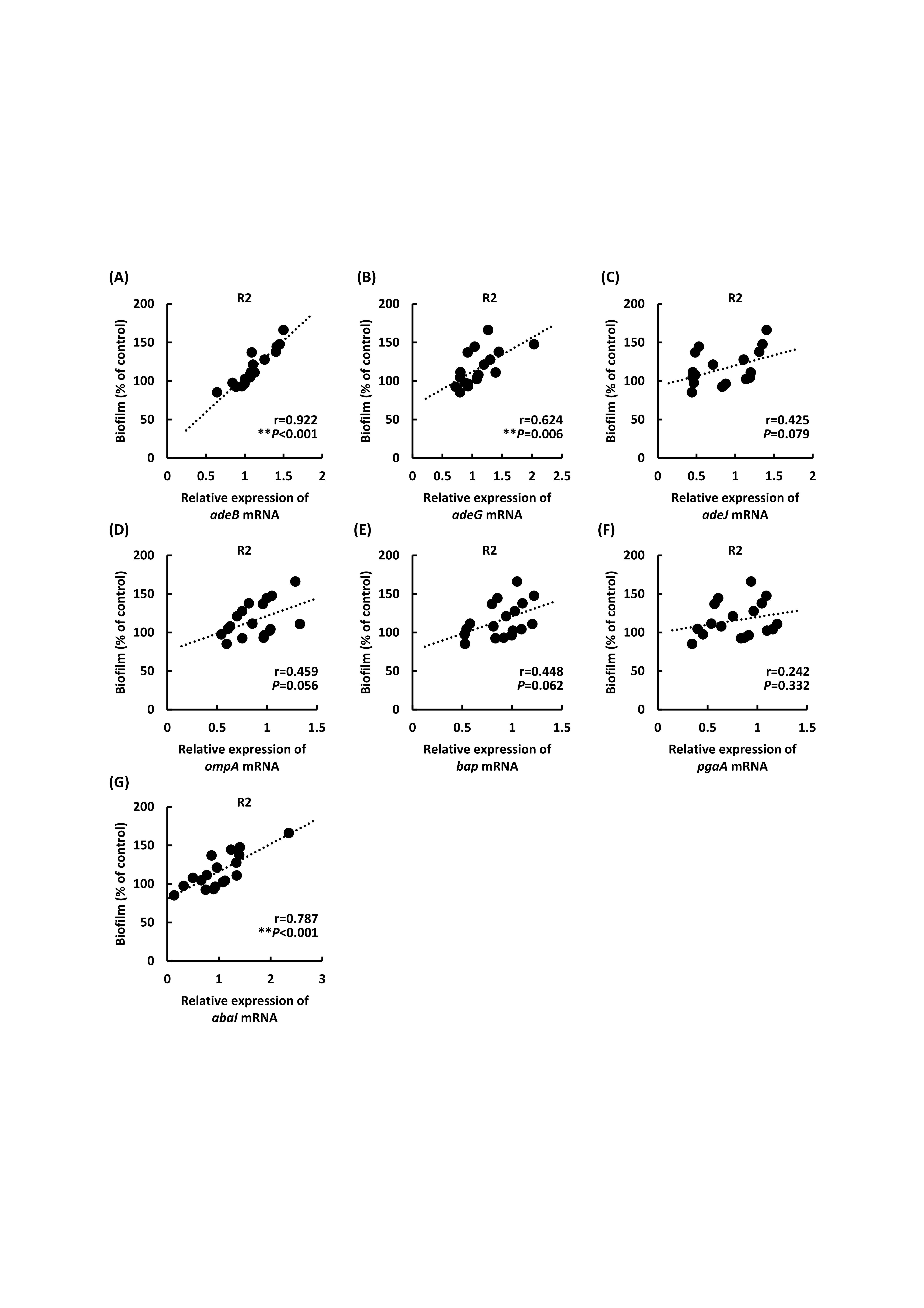

Supplement: S5 Fig — Pearson correlation coefficient was calculated for the number of biofilm cells and the expression of efflux pumps and biofilm-related genes in strain R2. (A) adeB mRNA (Pearson correlation coefficient r = 0.922, P<0.001), (B) adeG mRNA (Pearson correlation coefficient r = 0.624, P = 0.006), (C) adeJ mRNA (Pearson correlation coefficient r = 0.425, P = 0.079), (D) ompA mRNA (Pearson correlation coefficient r = 0.459, P = 0.056), (E) bap mRNA (Pearson correlation coefficient r = 0.448, P = 0.062), (F) pgaA mRNA (Pearson correlation coefficient r = 0.242, P = 0.332), and (G) abaI mRNA (Pearson correlation coefficient r = 0.787, P<0.001). Each symbol represents R2 strain in the absence and presence of CST. (TIF) [file pone.0194556.s006.tif]

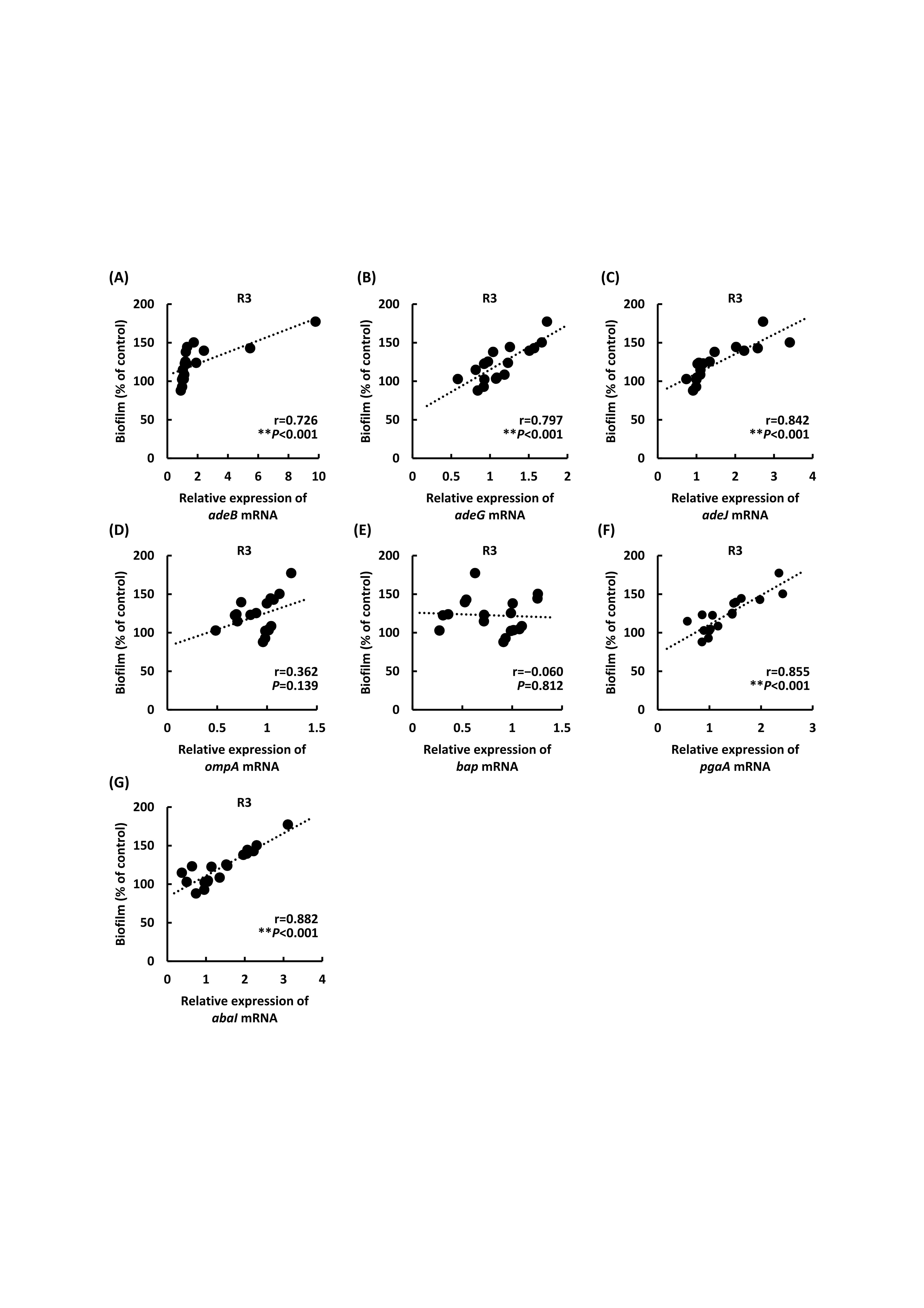

Supplement: S6 Fig — Pearson correlation coefficient was calculated for the number of biofilm cells and the expression of efflux pumps and biofilm-related genes in strain R3. (A) adeB mRNA (Pearson correlation coefficient r = 0.726, P<0.001), (B) adeG mRNA (Pearson correlation coefficient r = 0.797, P<0.001), (C) adeJ mRNA (Pearson correlation coefficient r = 0.842, P<0.001), (D) ompA mRNA (Pearson correlation coefficient r = 0.362, P = 0.139), (E) bap mRNA (Pearson correlation coefficient r = −0.060, P = 0.812), (F) pgaA mRNA (Pearson correlation coefficient r = 0.855, P<0.001), and (G) abaI mRNA (Pearson correlation coefficient r = 0.882, P<0.001). Each symbol represents R3 strain in the absence and presence of CST. (TIF) [file pone.0194556.s007.tif]

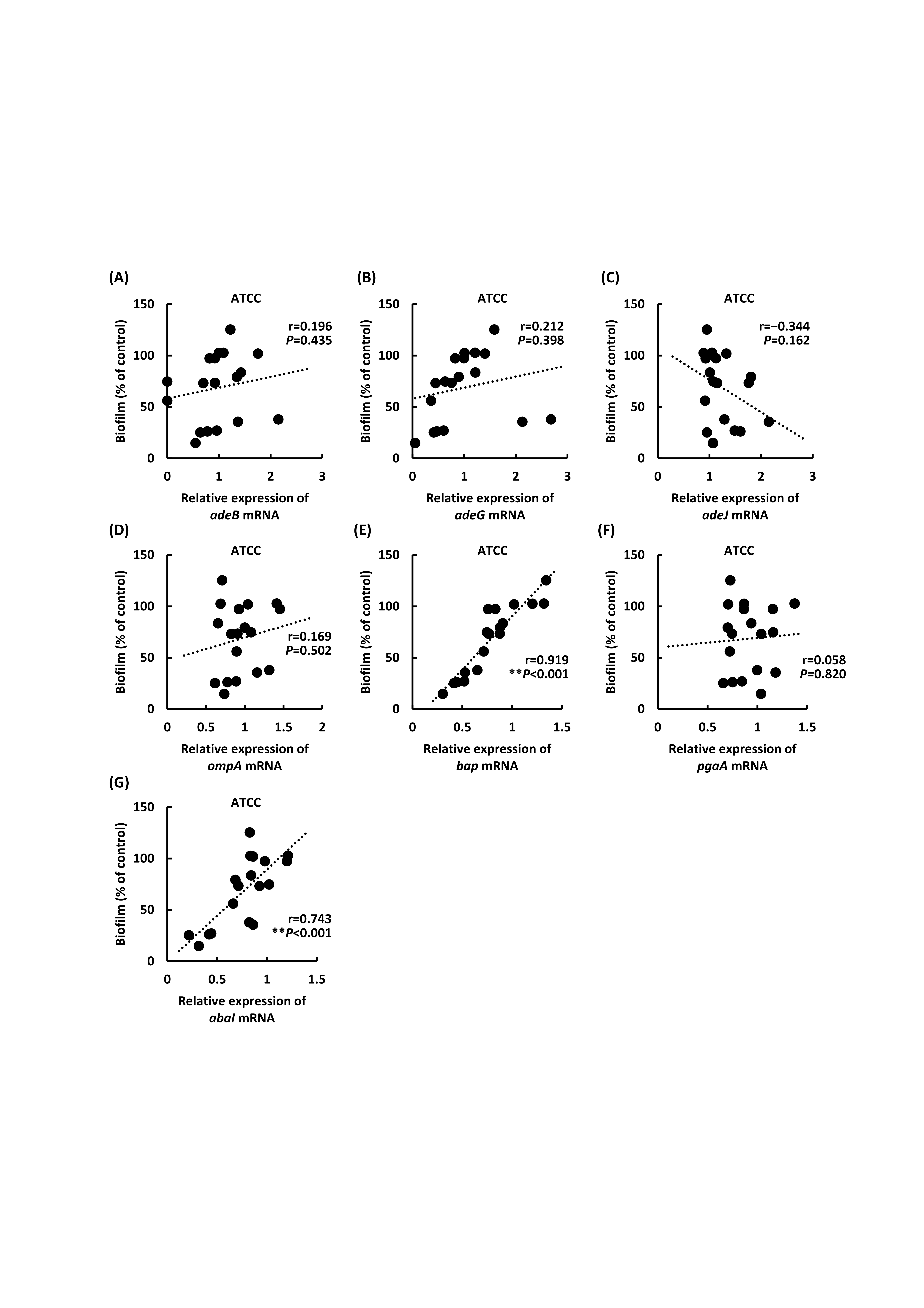

Supplement: S7 Fig — Pearson correlation coefficient was calculated for the number of biofilm cells and the expression of efflux pumps and biofilm-related genes in strain ATCC 19606. (A) adeB mRNA (Pearson correlation coefficient r = 0.196, P = 0.435), (B) adeG mRNA (Pearson correlation coefficient r = 0.212, P = 0.398), (C) adeJ mRNA (Pearson correlation coefficient r = −0.344, P = 0.162), (D) ompA mRNA (Pearson correlation coefficient r = 0.169, P = 0.502), (E) bap mRNA (Pearson correlation coefficient r = 0.919, P<0.001), (F) pgaA mRNA (Pearson correlation coefficient r = 0.058, P = 0.820), and (G) abaI mRNA (Pearson correlation coefficient r = 0.743, P<0.001). Each symbol represents ATCC19606 strain in the absence and presence of PMB. (TIF) [file pone.0194556.s008.tif]

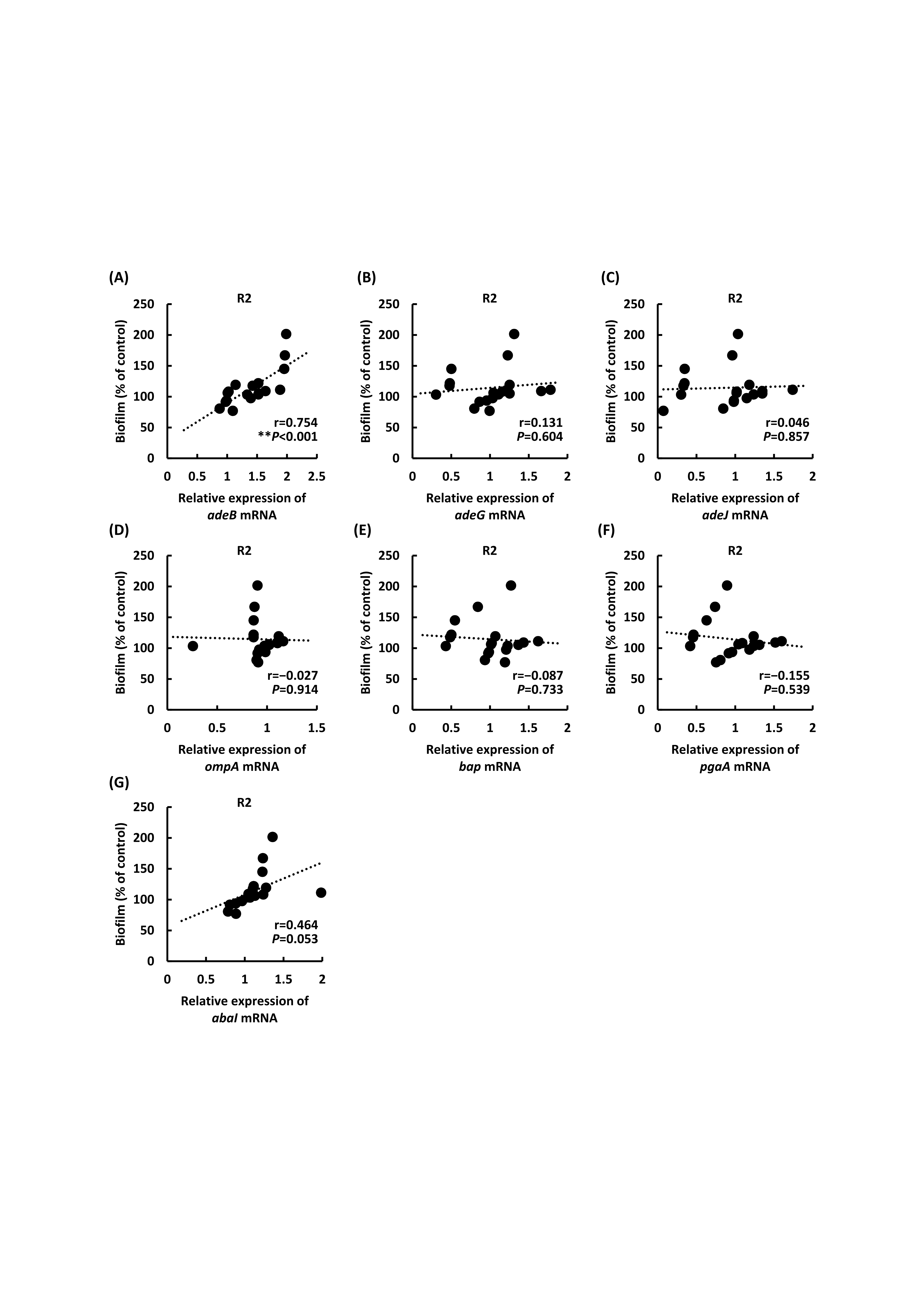

Supplement: S8 Fig — Pearson correlation coefficient was calculated for the number of biofilm cells and the expression of efflux pumps and biofilm-related genes in strain R2. (A) adeB mRNA (Pearson correlation coefficient r = 0.754, P<0.001), (B) adeG mRNA (Pearson correlation coefficient r = 0.131, P = 0.604), (C) adeJ mRNA (Pearson correlation coefficient r = 0.046, P = 0.857), (D) ompA mRNA (Pearson correlation coefficient r = −0.027, P = 0.914), (E) bap mRNA (Pearson correlation coefficient r = −0.087, P = 0.733), (F) pgaA mRNA (Pearson correlation coefficient r = −0.155, P = 0.539), and (G) abaI mRNA (Pearson correlation coefficient r = 0.464, P = 0.053). Each symbol represents R2 strain in the absence and presence of PMB. (TIF) [file pone.0194556.s009.tif]

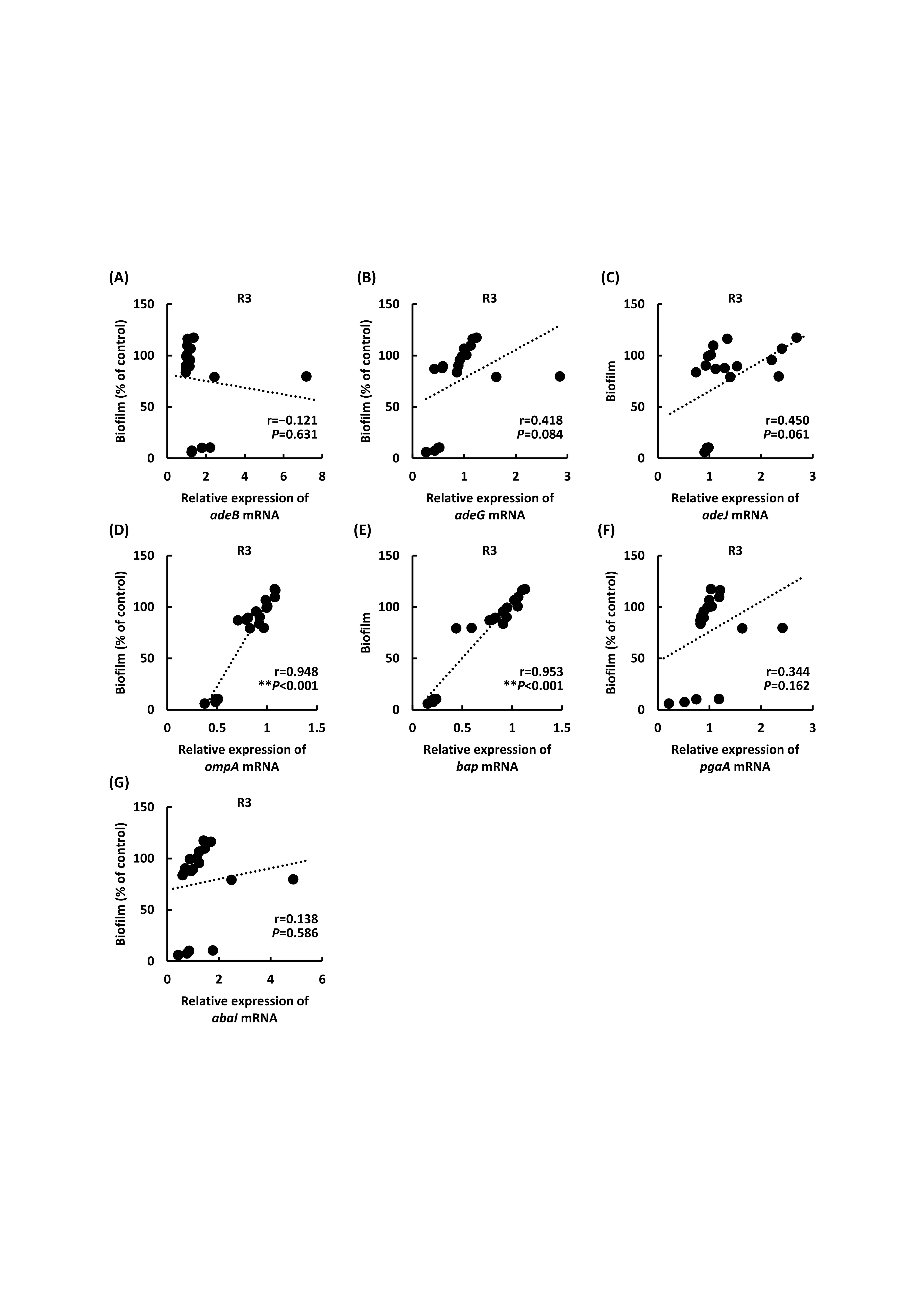

Supplement: S9 Fig — Pearson correlation coefficient was calculated for the number of biofilm cells and the expression of efflux pumps and biofilm-related genes in strain R3. (A) adeB mRNA (Pearson correlation coefficient r = −0.121, P = 0.631), (B) adeG mRNA (Pearson correlation coefficient r = 0.418, P = 0.084), (C) adeJ mRNA (Pearson correlation coefficient r = 0.450, P = 0.061), (D) ompA mRNA (Pearson correlation coefficient r = 0.948, P<0.001), (E) bap mRNA (Pearson correlation coefficient r = 0.953, P<0.001), (F) pgaA mRNA (Pearson correlation coefficient r = 0.344, P = 0.162), and (G) abaI mRNA (Pearson correlation coefficient r = 0.138, P = 0.586). Each symbol represents R3 strain in the absence and presence of PMB. (TIF) [file pone.0194556.s010.tif]
